# LncRNA UCA1 Induces Acquired Resistance to Gefitinib by Epigenetically Silencing CDKN1A Expression in Non-small-Cell Lung Cancer

**DOI:** 10.3389/fonc.2020.00656

**Published:** 2020-05-12

**Authors:** Tianwei Xu, Shuai Yan, Mengwei Wang, Lihua Jiang, Pei Ma, Binbin Lu, Qinnan Chen, Chenchen Wei, Zhaoxia Wang

**Affiliations:** ^1^Cancer Medical Center, The Second Affiliated Hospital of Nanjing Medical University, Nanjing, China; ^2^Department of Oncology, The Affiliated Jiangyin Hospital of Southeast University Medical College, Jiangyin, China; ^3^Department of Oncology, The First Affiliated Hospital of Nanjing Medical University, Nanjing, China

**Keywords:** lncRNA, UCA1, CDKN1A, NSCLC, gefitinib, resistance

## Abstract

Lung cancer is the most common cancer globally and is associated with high morbidity and mortality. Gefitinib has been widely used for treating advanced non-small-cell lung cancer (NSCLC). However, acquired resistance usually develops, although we still know little about the mechanism underlying this. In the present study, we found that the lncRNA UCA1 was upregulated in NSCLC tissues and cells with acquired gefitinib resistance, indicating the special role of UCA1 in gefitinib resistance. Knockdown of UCA1 promoted the sensitivity to gefitinib both *in vitro* and *in vivo* by suppressing cell proliferation and inducing apoptosis. Moreover, UCA1 could interact with EZH2 (enhancer of zeste homolog 2) to epigenetically reduce the expression of CDKN1A. Taking the obtained findings together, our study suggests that UCA1 is important for NSCLC to develop gefitinib resistance, and is a potential biomarker for gefitinib resistance and a therapeutic target for advanced NSCLC.

## Introduction

Cancer remains a major issue in healthcare and a serious threat to human health globally (1). Lung cancer is the leading cause of cancer-related death worldwide (2). Most patients are diagnosed at an advanced stage and, in terms of the treatment for lung cancer, drug therapy remains the most significant treatment option. Targeted drugs represented by epidermal growth factor receptor (EGFR) tyrosine kinase inhibitors (EGFR-TKIs) have shown great benefits in EGFR-mutated non-small-cell lung cancer (NSCLC) in recent years (3, 4). Guidelines including those of the National Comprehensive Cancer Network (NCCN) and European Society for Medical Oncology (ESMO) have recommended EGFR-TKIs, such as gefitinib and erlotinib, as first-line treatment for advanced EGFR-mutant patients (5, 6). Unfortunately, acquired resistance usually develops after a median of 10–14 months on EGFR-TKIs (7). The mechanisms by which resistance is acquired are complex. The most common is T790M, which was reported to account for ~50–60% of cases with acquired resistance to first-generation EGFR-TKIs. However, the mechanisms of resistance in the remaining cases are still poorly understood and require further investigation (8).

Long noncoding RNAs (lncRNAs) are defined as RNAs longer than 200 nucleotides that do not encode a protein product. They participate in various cancer-related biological processes, including the cell cycle, proliferation, apoptosis, and autophagy (9–13). Our group also found that the lncRNA SNHG17 promotes cell proliferation and migration in NSCLC (13). Recent reports also suggested that lncRNAs play key roles in resistance to EGFR-TKIs in EGFR-mutant NSCLC patients (14–16). For instance, overexpression of LINC00665 was shown to mediate acquired resistance to gefitinib by activating the PI3K/AKT pathway (17). In addition, downregulation of the lncRNA GAS5 reduced sensitivity to gefitinib treatment (18). The lncRNA UCA1 was first reported in a human bladder cancer cell line (19). Studies have demonstrated its overexpression in various cancers including NSCLC (20). UCA1 is also now known to play an oncogenic role and to participate in the chemo resistance of NSCLC (21). Moreover, transcriptomic analysis in our previous study (15) indicated that UCA1 may also be involved in gefitinib resistance. Cheng et al. (22) also reported similar results and indicated the activation of the AKT/mTOR pathway by western blot. However, no in-depth research was reported.

In this study, the level of UCA1 was measured in gefitinib-resistant and gefitinib-sensitive NSCLC patients and cells. Knockdown of UCA1 promoted gefitinib sensitivity both *in vitro* and *in vivo*. CDKN1A has been reported to be essential to gefitinib treatment (23). We also revealed for the first time that the mechanism underlying this may be the epigenetic silencing of CDKN1A expression by UCA1. Taking these findings together, UCA1 is a potential therapeutic target for NSCLC patients with acquired resistance to gefitinib.

## Materials and Methods

### Tissue Samples and Clinical Data Collection

A total of 73 advanced NSCLC patients from the First and Second Affiliated Hospital of Nanjing Medical University who had either EGFR exon 19 deletion (19DEL) or L858R were enrolled in this study. None of these patients had received chemotherapy or radiotherapy. Forty-four of them were from the NG group and the others were collected after the acquisition of resistance to EGFR-TKIs during target therapy (GR group). Our study was approved by the Research Ethics Committee of The Second Affiliated Hospital of Nanjing Medical University and written informed consent was obtained from all patients. All collected tissue samples were immediately snap-frozen in liquid nitrogen and stored at −80°C until required. The clinical information of the patients is summarized in Table 1.

**Table 1:** The clinic-pathological factors of 73 NSCLC patients.

**Clinical characteristics**	**NG Group (*n* = 44)**	**GR Group (*n* = 29)**
Sex
Male	24 (54.55%)	14 (48.28%)
Female	20 (45.45%)	15 (51.72%)
Age
≤ 65	32 (72.73%)	18 (62.07%)
>65	12 (27.27%)	11 (37.93%)
Histological classification
SCC (squamous cell carcinoma)	0 (0%)	0 (0%)
AD (adenocarcinoma or others)	44 (100%)	29 (100%)
TNM stage
IIIB	5 (11.36%)	3 (10.34%)
IV	39 (88.64%)	26 (89.66%)
EGFR mutation
19 DEL	28 (63.64%)	19 (65.52%)
L858R	16 (36.36%)	10 (34.48%)
Smoking
Smoker	25 (56.82%)	16 (55.17%)
Non-Smoker	19 (43.18%)	13 (44.83%)

### Cell Culture

The human lung adenocarcinoma cell line PC-9 (EGFR exon 19 deletion) was purchased from the Institute of Biochemistry and Cell Biology at the Chinese Academy of Sciences (Shanghai, China). The gefitinib-resistant cell line PC-9/GR was cultured in RPMI DMEM medium, containing 10% fetal bovine serum (FBS), antibiotics (100 U/mL penicillin and 100 mg/mL streptomycin) and gefitinib (1 μM/L) at 37°C in humidified incubators with 5% CO_2_.

### RNA Isolation and Quantitative Real-Time PCR Analyses

Total RNA was extracted from tissues or cultured cells with TRIzol reagent (Invitrogen, Carlsbad, CA, USA). The isolated RNA (1.0 mg) was reverse-transcribed to cDNA using random primers with the Prime-Script RT reagent kit (Takara, Dalian, China), in accordance with the manufacturer's instructions. Real-time PCR analyses were conducted using SYBR Green (Takara). The results were normalized to the expression of glyceraldehyde 3-phosphate dehydrogenase (GAPDH). Specific primer sequences are listed in Supplementary Table 1. All experiments were performed in triplicate. Our qRT-PCR results were analyzed and expressed relative to threshold cycle (CT) values, and then converted to fold changes.

### RNAi and Transfection

PC-9/GR cells were seeded into six-well plates and transfected with 10 mL of specific siRNA or negative control siRNA (si-NC) using Lipofectamine 2000 (Invitrogen, Shanghai, China). The target sequence for si-UCA1 is listed in Supplementary Table 1. Cells were harvested 48 h after transfection for quantitative real-time PCR and other experiments.

### *In vitro* Gefitinib Sensitivity and Colony Formation Assays

Cell proliferation was measured using Cell Proliferation Reagent Kit I (MTT) (Roche Applied Science, Basel, Switzerland). PC-9/GR cells transfected with si-UCA1 or si-NC were plated into 96-well plates at a density of 3 × 10^3^/well and incubated overnight. Subsequently, the cells were exposed to different concentrations of gefitinib (AstraZeneca, London, UK) for 72 h. Then, cell viability was assessed following the manufacturer's protocol. All experiments were repeated three times independently.

In the colony formation assay, a total of 800 si-UCA1 or si-NC PC-9/GR cells were placed in six-well plates maintained in medium containing 10% FBS and exposed to gefitinib for 24 h. Then, the drug was washed away and the medium was replaced every 4 days. After 2 weeks, the colonies were fixed with methanol and stained with 0.1% crystal violet (Sigma, St. Louis, MO, USA). Visible colonies were counted. Each experiment was performed in triplicate.

### Ethynyl Deoxyuridine (EdU) (Red)/DAPI (blue) Immunostaining Assay

In PC-9/GR cells, DNA newly synthesized after the indicated treatment was detected by EdU fluorescence staining, in accordance with the manufacturer's instructions (Click-iT® EdU Imaging Kit; Invitrogen). The cells, cultured in a well of a 24-well plate at a density of 30,000 cells per well, were labeled with 10 μM EdU and incubated for an additional 2 h before being fixed with 3.7% formaldehyde for 15 min at room temperature. The fixative was subsequently removed and the cells in each well were washed twice with 1 ml of 3% BSA in PBS. The BSA was removed and 1 ml of 0.5% Triton® X-100 (Sigma, San Francisco, CA, USA) in PBS was added to each well and incubated at room temperature for 20 min. After washing the cells in each well twice with 3% BSA in PBS, the cells were reacted with 500 μL of 1 × Click-iT® reaction cocktail for 30 min at room temperature in the dark. Subsequently, for nuclear staining, 1 ml of 1 × Hoechst 33342 solution (Sigma, San Francisco, CA, USA) was added to each well and incubated for 30 min at room temperature in the dark. The Hoechst 33342 solution was removed, and the EdU-labeled cells were counted using fluorescence microscopy (CKX41-F32FL; Olympus, Tokyo, Japan) and normalized to the total number of Hoechst-stained cells. Image-Pro Plus software (Version 6.0; Media Cybernetics, Bethesda, MD, USA) was used to calculate the percentage of EdU-positive cells.

### Transwell Migration Assay

After the indicated treatment in serum-free RPMI DMEM, 5 × 10^4^ cells were seeded in the upper chamber (8 mm; Millipore), and RPMI DMEM containing 10% FBS was added to the lower chamber. After culturing for 24 h, the cells that had migrated through the membrane were fixed with methanol and stained with 0.1% crystal violet. Images were taken using an IX7 inverted microscope (Olympus, Tokyo, Japan). All experiments were conducted in triplicate.

### Flow Cytometric Analysis of Apoptosis and Cell Cycle

The PC-9/GR cells transfected with si-UCA1 or si-NC were treated with gefitinib for 72 h. Then, the cells were harvested by trypsinization and double-stained with fluorescein isothiocyanate (FITC)-Annexin V and propidium iodide using the FITC Annexin V apoptosis detection kit (BD Biosciences). The proportion of cells undergoing apoptosis was analyzed using a flow cytometer (FACScan; BD Biosciences, Shanghai, China). The BD Cycle Test Plus DNA Reagent Kit (BD Biosciences) was used in the cell-cycle analysis following the manufacturer's protocol. The proportions of cells in G_0_/G_1_, S, and G_2_/M phases were estimated. Each experiment was conducted three times independently.

### Tumor Formation Assay in Nude Mouse Model

Male athymic BALB/c nude mice (5 weeks old) were maintained under specific pathogen-free conditions and manipulated in accordance with protocols approved by the Shanghai Medical Experimental Animal Care Commission. LV-UCA1 and LV-control were purchased from Shanghai GENECHEM. PC-9/GR cells were transfected with LV-UCA1 or LV-control following the manufacturer's instructions. Cells with stable knockdown of UCA1 and control cells were suspended in PBS at a concentration of 2 × 10^7^ cells/mL and injected into either side of the posterior flank of mice in a volume of 100 mL. Nine days after inoculation, gefitinib treatment was administered by oral gavage 5 days per week at 25 mg/kg. The tumor volumes were measured every 3 days. Twenty days later, the tumors were resected from all mice and used for immunohistochemical (IHC) staining. The quantification of Ki-67 protein level was decided by two skilled pathologists independently.

### Western Blot Analysis and Antibodies

The total cellular protein lysates were separated by 10% SDS-PAGE and transferred to polyvinylidene fluoride (PVDF) membranes (Millipore, USA). The membranes were incubated with specific antibodies overnight at 4°C. GAPDH was used as an internal control. Anti-P21 and anti-EZH2 were purchased from Cell Signaling Technology (Beverly, MA, USA).

### Subcellular Fractionation Localization

The separation of nuclear and cytosolic fractions was performed using the PARIS Kit (Life Technologies, Carlsbad, CA, USA), in accordance with the manufacturer's instructions.

### RIP Assay

RIP experiments were performed using a Magna RIP RNA-binding protein immunoprecipitation kit (Millipore, Billerica, MA, USA), following the manufacturer's instructions. In brief, PC-9/GR cells were scraped off the culture plate and then lysed in RIP lysis buffer. Cell extract was incubated with RIP buffer containing magnetic beads conjugated with anti-EZH2, anti-SUZ12, or control immunoglobulin G (IgG) (Millipore). Finally, immunoprecipitated RNA was isolated and analyzed by quantitative real-time PCR.

### Chromatin Immunoprecipitation Assays

PC-9/GR cells were treated with formaldehyde and incubated for 10 min to generate DNA–protein crosslinks. Cell lysates were then sonicated to generate chromatin fragments of 200–300 bp and immunoprecipitated with EZH2- or H3K27me3-specific antibodies (Millipore) or IgG as a control. Precipitated chromatin DNA was recovered and analyzed by qRT-PCR.

### Statistical Analysis

All statistical analyses were performed using SPSS software package 22.0 (IBM, SPSS, USA) and GraphPad Prism 6 (GraphPad Software, La Jolla, CA, USA). Results are expressed as mean ± SD. The significance of differences between the different groups was analyzed by Student's *t*-test as appropriate. A *p* < 0.05 was considered statistically significant.

## Results

### Overexpression of UCA1 Is Correlated With Acquired Resistance to Gefitinib

**Figure 1:**
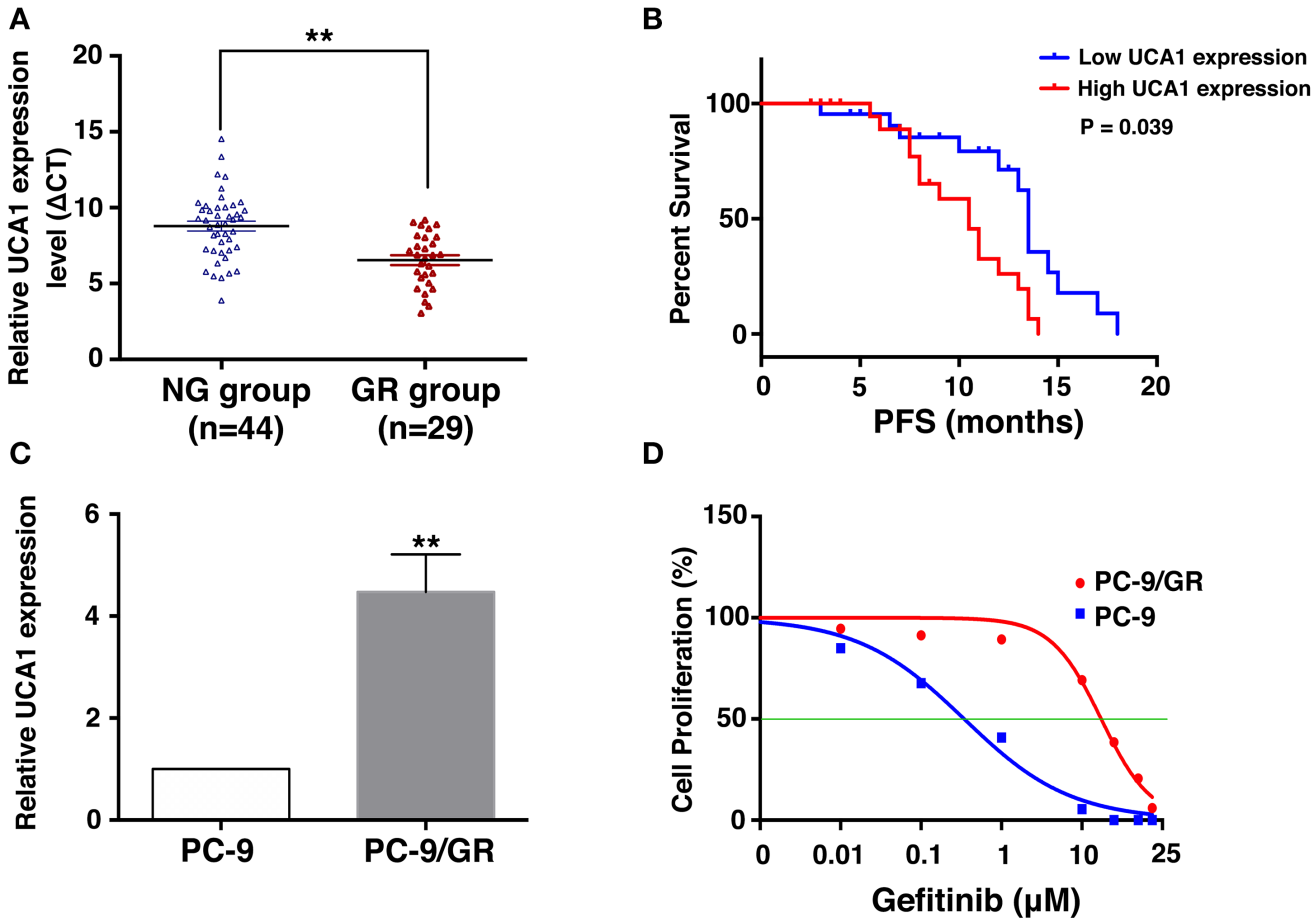
Expression of UCA1 in gefitinib-resistant tissues and cells and its clinical significance. **(A)** UCA1 expression in lung cancer tissues from patients who had never been treated with gefitinib (NG group) compared with patients who were treated with gefitinib and developed resistance (GR group) was measured by quantitative real-time PCR and normalized to GAPDH expression. **(B)** Progression-free survival (PFS) in NG group divided by high and low UCA1 expression levels before EGFR-TKI treatment. **(C)** UCA1 expression was analyzed by qPCR in gefitinib-sensitive PC-9 cell line and gefitinib-resistant PC-9/GR cell line. **(D)** IC50 of PC-9 and PC-9/GR cell lines were detected by MTT after various concentration of gefitinib treatment for 72 h (**p < 0.01).

To uncover the role of UCA1 in acquired resistance to gefitinib, we measured the expression of UCA1 in biopsy specimens of EGFR-mutant NSCLC patients. The clinicopathological characteristics of these patients are summarized in Table 1. The patients were divided into two groups: those who had never been treated with gefitinib (NG, *n* = 44) and those who had developed acquired resistance after gefitinib treatment (GR, *n* = 29). The expression level of UCA1 was significantly higher in the GR group than in the NG group (Figure 1A).

On the basis of UCA1 expression before gefitinib treatment, the patients were divided into a high-expression group and a low-expression group by the median level of UCA1 expression (Supplementary Figure 1). Progression-free survival (PFS) upon gefitinib treatment was plotted according to UCA1 expression levels. The results showed that patients with high UCA1 expression levels had poorer PFS (mPFS: 10.0 vs. 16.2 months, *P* = 0.039, Figure 1B). These findings indicate that UCA1 overexpression may represent a novel indicator of poor prognosis or a progression marker for gefitinib treatment.

To investigate the functional role of UCA1 in NSCLC gefitinib-resistant cells, we first determined its expression in gefitinib-sensitive cells (PC-9) and gefitinib-resistant cells (PC-9/GR). UCA1 was detected in both of these cell types, but its expression level was ~4.4-fold higher in PC-9/GR cells than that in PC-9 cells (Figure 1C). The gefitinib resistance of PC-9/GR was confirmed by MTT (Figure 1D). Thus, high expression levels of UCA1 may correlate with gefitinib resistance.

### UCA1 Inhibition Partially Restores Gefitinib Sensitivity *in vitro*

**Figure 2:**
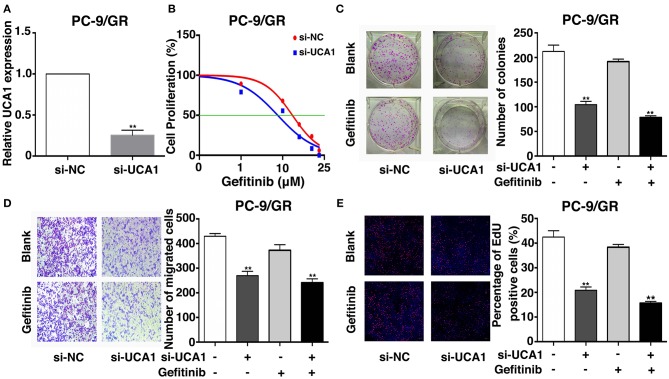
Effects of UCA1 on gefitinib resistance *in vitro*. **(A)** The expression levels of UCA1 was knockdown by si-UCA1 in PC-9/GR. **(B)** IC50 of transfected PC9-R/GR cells were detected by MTT after various concentration of gefitinib treatment for 72 h. **(C)** Colony-forming assays were performed to determine the proliferation of transfected PC-9/GR cells treated with or without gefitinib. **(D)** PC-9/GR cells were analyzed by Transwell assays 48 h after transfection. **(E)** Proliferous transfected PC-9/GR cells were displayed by EdU immunostaining assays treated with or without gefitinib (**p < 0.01).

To determine the role of UCA1 in gefitinib resistance, the expression of UCA1 was downregulated by 75% using si-UCA1 (Figure 2A). This UCA1 knockdown significantly reduced the IC_50_ of PC-9/GR cells treated with gefitinib (Figure 2B). Colony formation assay showed that the knockdown of UCA1 decreased the colony-forming ability of PC-9/GR cells in the presence of gefitinib (Figure 2C). Ethynyl deoxyuridine (EdU) (red)/DAPI (blue) immunostaining assay also confirmed the above results (Figure 2E). Transwell assay indicated that UCA1 knockdown plus gefitinib decreased cell migration. However, similar changes were not observed with gefitinib alone (Figure 2D). Taken together, these results suggest that UCA1 knockdown can promote the sensitivity of PC-9/GR to gefitinib *in vitro*.

### Knockdown of UCA1 Induces Apoptosis and Promotes Cell-Cycle Arrest

**Figure 3:**
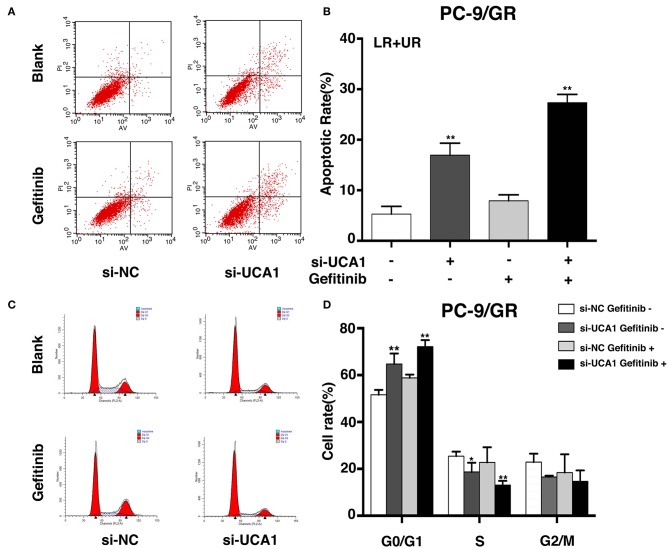
UCA1 knockdown induced apoptosis and arrested cell cycle at the G0/G1 phase in PC-9/GR cells under gefitinib treatment *in vitro*. **(A,B)** Apoptotic rates of PC-9/GR cells 48 h after transfection were detected by flow cytometry assays. **(C,D)** Cell cycle stages of PC-9/GR cells after the indicated treatment were analyzed (*p < 0.05 and **p < 0.01).

To examine whether the effect of UCA1 knockdown in gefitinib-resistant cells depends on apoptosis or cell-cycle progression, flow cytometric analysis was performed. The proportion of apoptotic cells significantly increased following treatment with si-UCA1 and gefitinib together (Figures 3A,B). In addition, the knockdown of UCA1 in PC-9/GR cells promoted cell-cycle arrest at the G_1_-G_0_ phase and reduced the number of cells in the G_2_-S phase (Figures 3C,D). These findings indicate that UCA1 may drive gefitinib resistance in NSCLC by inhibiting apoptosis and the G_1_-S checkpoint.

### Downregulation of UCA1 Inhibits Gefitinib-Resistant NSCLC Development *in vivo*

**Figure 4:**
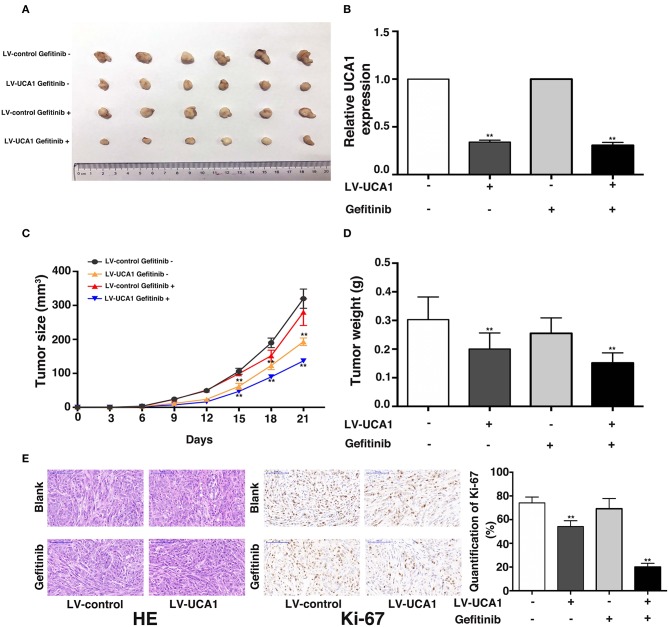
Knockdown of UCA1 overcame acquired resistance to gefitinib *in vivo*. **(A)** PC-9/GR cells treated with LV-control or LV-UCA1 were injected into nude mice. 9 days later, the mice were treated with blank control or gefitinib. **(B)** qRT-PCR was performed to detect the average expression of UCA1 in xenograft tumors **(C)** Tumor volumes were calculated every 3 days **(D)** Tumor weight was measured after removal. **(E)** Tumors developed from LV-UCA1-PC-9/GR cells with gefitinib treatment showed lower Ki-67 protein levels (**p < 0.01).

We used a xenograft mouse model to further investigate the role of UCA1 in gefitinib resistance. PC-9/GR cells stably transfected with LV-UCA1 or LV-control were inoculated into male nude mice. Twenty-one days after injection, all mice developed xenograft tumors at the injection site. As expected, gefitinib with LV-control modestly inhibited tumor growth. The repression of UCA1 significantly reduced tumor growth (Figures 4A–D). As shown in Figure 4E, immunohistochemistry confirmed that tumors formed from PC-9/GR/LV-UCA1 cells displayed lower-intensity Ki-67 staining than those formed from empty vector-transfected cells in the presence of gefitinib. These results suggested that UCA1 contributes to gefitinib-resistant tumorigenesis *in vivo*.

### UCA1 Drives Gefitinib Resistance via Epigenetically Silencing CDKN1A Transcription

**Figure 5:**
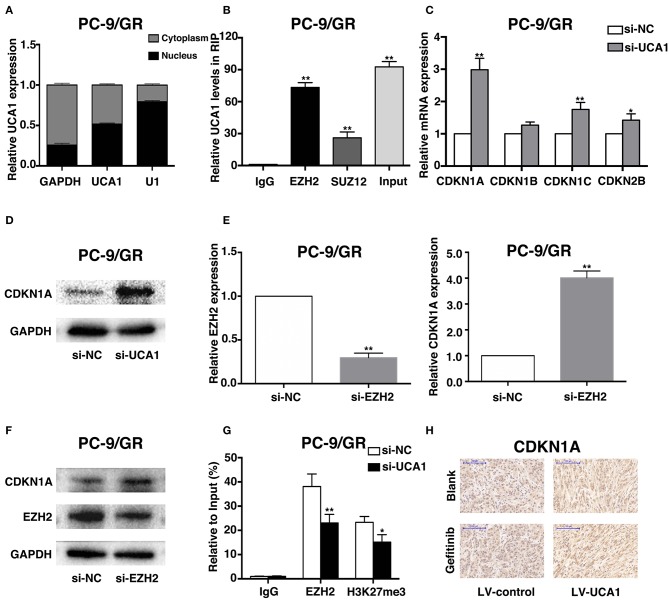
UCA1 Epigenetically silences CDKN1A transcription by binding to EZH2. **(A)** UCA1 expression levels in the cytoplasm or nucleus of PC-9/GR were detected by qRT-PCR. GAPDH was used as a cytosol marker and U1 was used as a nuclear marker. **(B)** RIP experiments were performed in PC-9/GR and the coprecipitated RNA was subjected to qRT-PCR for UCA1. UCA1 RNA expression levels are presented as fold enrichment in EZH2 and SUZ12 immunoprecipitated relative to that of IgG. **(C)** The expression of CDKN1A, CDKN1B, CDKN1C, and CDKN2B was determined using qRT-PCR after knockdown of UCA1. **(D)** Western blot analysis was conducted to detect the level of CDKN1A protein in PC-9/GR transfected with si-UCA1. **(E,F)** qRT-PCR and western blot assays were used to detect EZH2 and CDKN1A mRNA and protein levels in PC-9/GR cells transfected with si-EZH2. **(G)** ChIP-qRT-PCR of EZH2 occupancy and H3K27me3 binding to the CDKN1A promoter in PC-9/GR treated with si-UCA1 or si-NC; IgG as a negative control. **(H)** Upregulation of CDKN1A expression after UCA1 knockdown was also confirmed *in vivo* by immunohistochemistry (^*^p < 0.05 and **p < 0.01).

To explore the molecular mechanisms by which UCA1 contributes to gefitinib resistance, we first determined the subcellular localization of UCA1 in PC-9/GR using fractionation assays. UCA1 was present in both the cytoplasm and the nucleus of PC-9/GR (Figure 5A), indicating that it may also act as a transcriptional regulator in the nucleus, besides its well-known role as a sponge in the cytoplasm (21). Nearly 20% of human lncRNAs are bound by the polycomb repressive complex 2 (PRC2), thereby modulating the expression of downstream targets (24). EZH2 and SUZ12, two core subunits of PRC2, were selected to perform RNA immunoprecipitation (RIP) assays. The results showed that UCA1 binds directly to EZH2 in PC-9/GR (Figure 5B).

To identify the downstream genes involved in gefitinib resistance, we determined the differentiated expression of potential targets of EZH2, including CDKN1A, CDKN1B, CDKN1C, and CDKN2B. These genes have also been reported to be associated with gefitinib activities (25–27). qRT-PCR results showed that the knockdown of UCA1 significantly increased CDKN1A expression compared with that in control cells (Figure 5C). Moreover, clear upregulation of the level of CDKN1A was identified by qPCR and western blot after the silencing of UCA1 and EZH2 (Figures 5D–F; Supplementary Figure 2).

**Figure 6:**
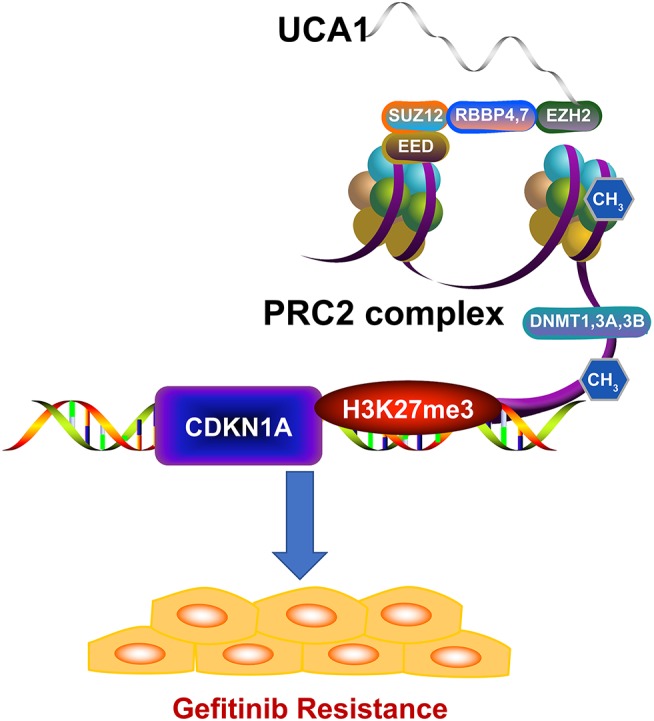
Model of UCA1 function and mechanisms during gefitinib resistance. UCA1 promoted gefitinib resistance through epigenetically silencing CDKN1A transcription by binding to EZH2 (The structure of PRC2 complex was downloaded from Reactome^28^).

We next investigated whether EZH2 could bind to the promoter regions of CDKN1A, mediated by UCA1. For this, we performed chromatin immunoprecipitation (ChIP) assays. The results showed that EZH2 could bind to the CDKN1A promoter regions, and that the downregulation of UCA1 reduced EZH2-mediated H3K27me3 trimethylation (Figure 5G). Finally, upregulation of CDKN1A expression after UCA1 knockdown was also confirmed *in vivo* by immunohistochemistry (Figure 5H). These results suggest that the lncRNA UCA1 could promote gefitinib resistance through epigenetically silencing CDKN1A transcription by binding to EZH2 (Figure 6) (28).

## Discussion

Recently, numerous studies have revealed the specific role of lncRNAs in tumorigenesis, such as proliferation and migration (29). Some studies also focused on the relationship between lncRNAs and treatment resistance in lung cancer (14, 16). Our previous study also demonstrated that downregulation of the lncRNA MEG3 contributes to cisplatin resistance of lung adenocarcinoma (30). In addition, it was shown that HOTAIR promotes this cisplatin resistance by silencing CDKN1A (31). In our study, we demonstrated that the lncRNA UCA1 contributed to the acquisition of resistance to gefitinib. High expression of UCA1 was found to be associated with a shorter PFS upon gefitinib treatment. Moreover, mechanistic experiments identified CDKN1A as the target of UCA1 for the development of gefitinib resistance. PRC2 is a critical regulator of histone modification to mediate gene silencing by promoting the trimethylation of H3K27 (32). EZH2 is a core subunit of the PRC2 complex, which can promote the trimethylation of H3K27me3 (33). In our study, RIP assays confirmed that UCA1 could bind to EZH2 and ChIP assays confirmed that CDKN1A was a bona fide target of UCA1/EZH2-regulated genes.

Gefitinib is an EGFR-targeting small molecule that has been proposed as the first-line treatment for EGFR-mutant NSCLC patients. However, the emergence of acquired resistance is almost inevitable, but we still know little about the molecular mechanisms behind this. Our group previously used transcriptomic analysis to demonstrate the complex alterations of lncRNA expression in gefitinib resistance and indicate the important roles of lncRNAs in its emergence (15). Our study is the first to prove that the lncRNA UCA1 can interact with EZH2 to reduce CDKN1A expression, thereby leading to gefitinib resistance. CDKN1A is known as a critical cell-cycle checkpoint protein and is closely associated with drug resistance in cancer (34). Zhao et al. (23) reported that the silencing of CDKN1A led to acquired resistance to gefitinib and that its restoration promoted sensitivity to gefitinib by inducing cytostasis. In addition, Ji et al. (35) found that combined treatment with TNF-α/gefitinib alleviated gefitinib resistance by modulating the expression of CDKN1A. Polyphyllin VII could upregulate CDKN1A expression in gefitinib-resistant cells to elevate the sensitivity to gefitinib (36). These studies demonstrated that the regulation of CDKN1A is a potential therapy to reverse the acquisition of resistance to gefitinib. UCA1 could be a novel therapy target in drug resistance (37) by modulating the expression of CDKN1A. Transcription factors such as SATB1 could be used to directly knock down UCA1 expression (38). Moreover, RNA targeted therapeutics such as RNAi and ASO have been confirmed to be efficacious against cancer in clinical trials (39). The UCA1/EZH2/CDKN1A axis has potential in clinical applications aimed at reversing gefitinib resistance.

In summary, our findings show that UCA1 overexpression is associated with poor prognosis of NSCLC patients with acquired resistance to gefitinib. UCA1 may promote gefitinib resistance through silencing CDKN1A expression. Better understanding of UCA1/EZH2/CDKN1A axis may be helpful for the reverse of EGFR-TKI resistance. Our study provides a new perspective on UCA1 acting as a noncoding oncogene in NSCLC drug resistance. Therefore, it can become a novel target for overcoming the acquired gefitinib resistance in NSCLC.

## Data Availability Statement

All datasets generated for this study are included in the article/Supplementary Files.

## Ethics Statement

The animal study was reviewed and approved by Research Ethics Committee of Nanjing Medical University. The studies involving human participants were reviewed and approved by Research Ethics Committee of The Second Affiliated Hospital of Nanjing Medical University. The patients/participants provided their written informed consent to participate in this study.

## Author Contributions

Conception and design: ZW and TX. Development of the methodology: MW, SY, LJ, and QC. Acquisition of data: MW, BL, and CW. Writing the manuscript: TX and SY. Administrative, technical, and material support: ZW and PM. All authors read and approved the final manuscript.

## Conflict of Interest

The authors declare that the research was conducted in the absence of any commercial or financial relationships that could be construed as a potential conflict of interest.
