# Supplementary material for: LncRNA UCA1 Induces Acquired Resistance to Gefitinib by Epigenetically Silencing CDKN1A Expression in Non-small-Cell Lung Cancer
Source: Front Oncol. 2020 May 12;10:656. doi: 10.3389/fonc.2020.00656 (PMC7235350; doi:10.3389/fonc.2020.00656)
Supplement: Supplementary file 1 [file Data_Sheet_1.PDF]

Supplementary Figure 1

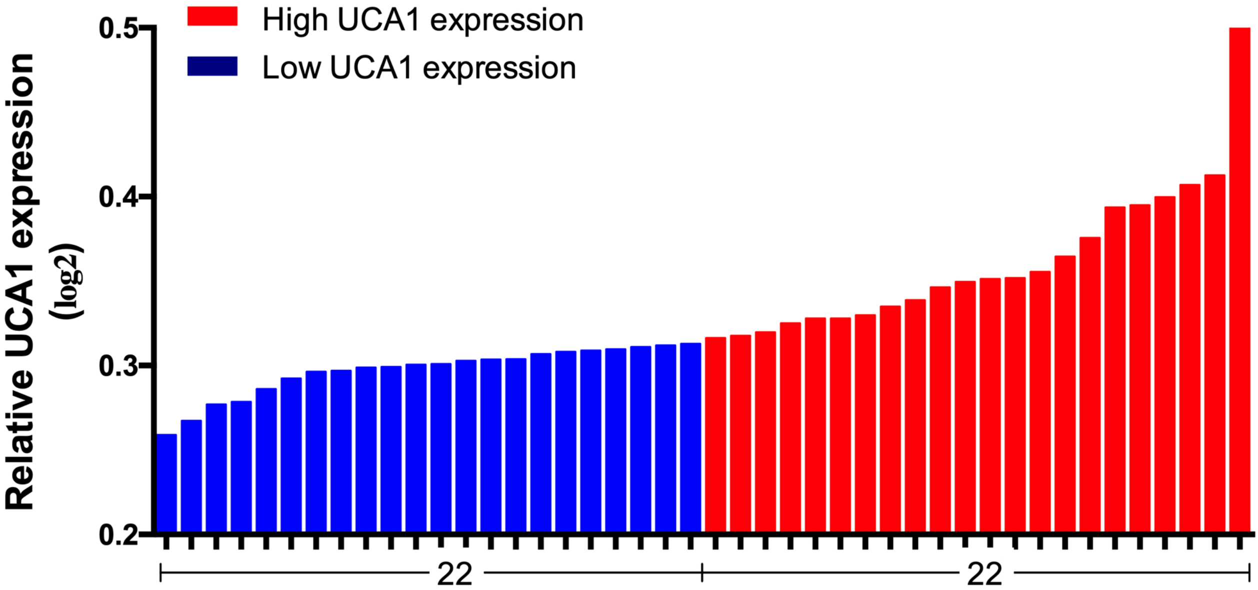

Patients were divided into two groups by the median of the log2 of ΔCT value

Supplementary Figure 2

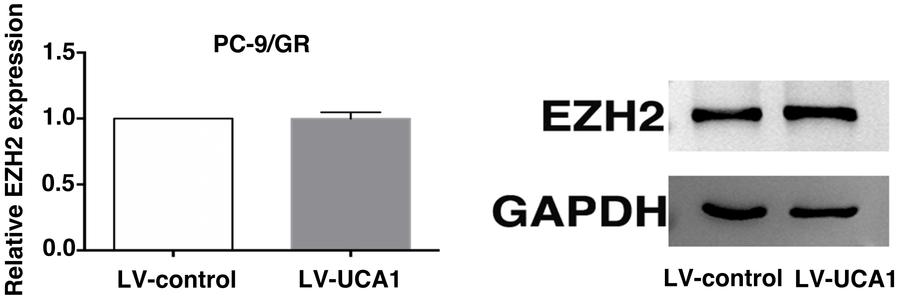

The LV-UCA1 can't itself downregulate EZH2

Supplementary Tables

qPCR primers

GAPDH F	GGGAGCCAAAAGGGTCAT
GAPDH R	GAGTCCTTCCACGATACCAA
UCA1 F	CTCTCCATTGGGTTCACCATTC
UCA1 R	GCGGCAGGTCTTAAGAGATGAG
CDKN1A F	AAGTCAGTTCCTTGTGGAGCC
CDKN1A R	GGTTCTGACGGACATCCCCA
CDKN1B F	TGCAACCGACGATTCTTCTACTCAA
CDKN1B R	CAAGCAGTGATGTATCTGATAAACAAGG
CDKN1C F	CACGATGGAGCGTCTTGTC
CDKN1C R	CCTGCTGGAAGTCGTAATCC
CDKN2B F	CTAGTGGAGAAGGTGCGACAG
CDKN2B R	CATCATCATGACCTGGATCGC

ChIP-qRT-PCR primers

CDKN1A F	GGTGTCTAGGTGCTCCAGGT
CDKN1A R	GCACTCTCCAGGAGGACACA

siRNA sequence

si-UCA1	AACUGGCACCUUGUUAGCUACAUAA
	UUAUGUAGCUAACAAGGUGCCAGUU
si-EZH2	GAGGUUCAGACGAGCUGAUUU
	AUCAGCUCGUCUGAACCUCUU
